# Neurofilament Phosphorylation during Development and Disease: Which Came First, the Phosphorylation or the Accumulation?

**DOI:** 10.1155/2012/382107

**Published:** 2012-04-18

**Authors:** Jeffrey M. Dale, Michael L. Garcia

**Affiliations:** ^1^Division of Biological Sciences, University of Missouri-Columbia, Columbia, MO 65211, USA; ^2^Bond Life Sciences Center, University of Missouri-Columbia, Columbia, MO 65211, USA

## Abstract

Posttranslational modification of proteins is a ubiquitous cellular mechanism for regulating protein function. Some of the most heavily modified neuronal proteins are cytoskeletal proteins of long myelinated axons referred to as neurofilaments (NFs). NFs are type IV intermediate filaments (IFs) that can be composed of four subunits, neurofilament heavy (NF-H), neurofilament medium (NF-M), neurofilament light (NF-L), and **α**-internexin. Within wild type axons, NFs are responsible for mediating radial growth, a process that determines axonal diameter. NFs are phosphorylated on highly conserved lysine-serine-proline (KSP) repeats located along the C-termini of both NF-M and NF-H within myelinated axonal regions. Phosphorylation is thought to regulate aspects of NF transport and function. However, a key pathological hallmark of several neurodegenerative diseases is ectopic accumulation and phosphorylation of NFs. The goal of this review is to provide an overview of the posttranslational modifications that occur in both normal and diseased axons. We review evidence that challenges the role of KSP phosphorylation as essential for radial growth and suggests an alternative role for NF phosphorylation in myelinated axons. Furthermore, we demonstrate that regulation of NF phosphorylation dynamics may be essential to avoiding NF accumulations.

## 1. Introduction

**Figure 1:**
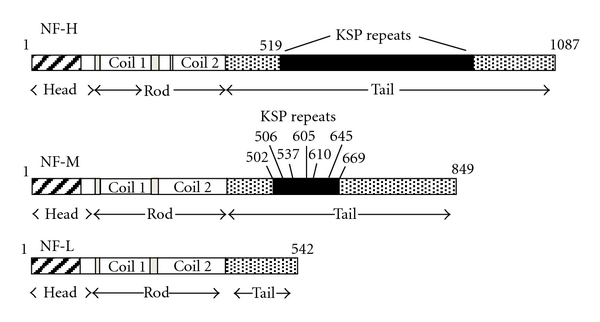
Representation of the functional domains of each NF subunit. The subdomains within each domain are identified along with approximate location of all relevant amino acid positions.

The established role of neurofilaments (NFs) is to increase axonal diameter in myelinated fibers thereby increasing nerve conduction velocity [1]. NFs are composed of an N-terminal head, central rod, and C-terminal tail domain [2] (Figure 1). NFs are posttranslationally modified in all three of these functional domains [3–7]. NF phosphorylation, the most frequent posttranslational modification (PTM) and focus of our review, occurs primarily at conserved KSXXP motifs (KSP) located on the C-terminal tail domain of neurofilament heavy (NF-H) and medium (NF-M) [3–6]. However, phosphorylation of “non-KSP” serine residues within NF-M and neurofilament light (NF-L) amino terminal (N-terminal) head domain has been observed [7]. Transgenic [8] and gene-targeted [9] mouse lines expressing mutagenized NF phosphorylation sites have significantly advanced our understanding of the role of NF phosphorylation. For example, mice expressing a serine to aspartate mutation at position 55 of NF-L, NF-L^S55D^, displayed accumulations of phosphorylated NFs within cell bodies [8]. Furthermore, mice expressing serine to alanine mutations within the 7 identified KSP motifs of NF-M, NF-M^S→A^, demonstrated an unaltered distribution of axonal diameters [9].

 Over the last 20 years, great strides have been taken in the characterization of NF proteins, their function, and their PTMs. In this review, we will attempt to describe the experiments that have led to the current understanding of the role of NF proteins and their PTMs in normal and diseased axons. Furthermore, using other intermediate filaments as a reference, we will detail a potential role for NF phosphorylation. Moreover, we will address the importance of investigating this putative role of NF phosphorylation and its potential to explain abnormal NF accumulations in neurodegenerative diseases.

## 2. Does KSP Phosphorylation Regulate Axon Diameter?

Axonal diameter influences the rate of neuronal conduction velocity with larger caliber axons conducting faster than small caliber axons [10–12]. In myelinated axons, the internode (myelinated) has a larger diameter than nodes of Ranvier (unmyelinated). Axonal diameter is established by a process referred to as radial axonal growth. Axonal NFs were required for radial growth [13, 14]. NF phosphorylation and radial growth were reduced in axons when compact myelin failed to form [15]. Moreover, phosphorylation of NFs C-termini was increased in myelinated axonal regions relative to unmyelinated regions of the same axon [16, 17]. Taken together, these data suggested that KSP repeat phosphorylation was required for establishing axonal diameter.

**Figure 2:**
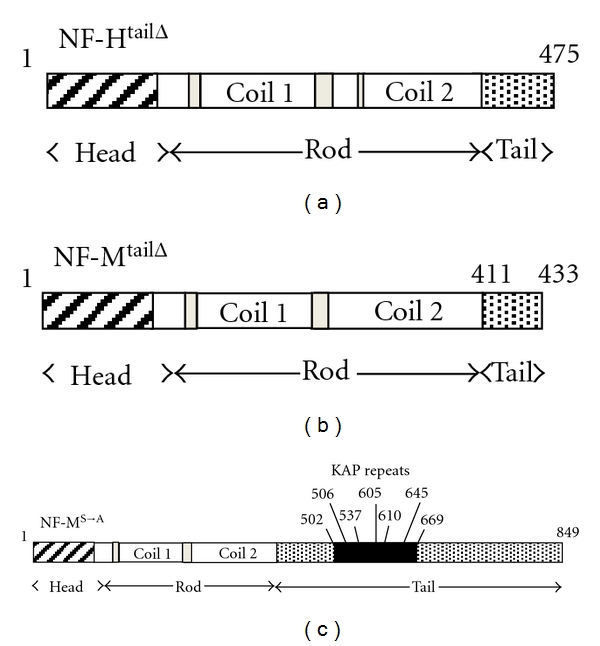
Representation of NF-H and NF-M mutants. (a) Incorporation of a C-terminal myc tag results in loss of 612 amino acids including all KSP repeat motifs. (b) Incorporation of a C-terminal myc tag results in loss of 426 amino acids including 7 KSP repeat motifs. (c) Site-directed mutagenesis was utilized to mutate all KSP serine residues to alanine thereby preventing KSP serine phosphorylation without deletion of the remaining amino acids. The approximate location of each KSP repeat is identified.

 Deletion of NF-M [18, 19] resulted in a reduction in radial growth that was similar to nerves without axonal NFs [14], whereas deletion of NF-H [20–22] did not reduce axonal diameter to a similar level as loss of NF-L [14] or NF-M [18, 19]. Moreover, axonal diameter was reduced in mice expressing C-terminally truncated NF-M (NF-M^TailΔ^) or C-terminally truncated NF-M and NF-H (NF-(M/H)^TailΔ^) [23] (Figures 2(a) and 2(b)). However, truncation of NF-H (NF-H^TailΔ^) only reduced axonal diameter in young mice [22]. Axonal diameters in older NF-H^TailΔ^ mice were similar to wild type [22]. These data suggested that NF-M and its C-terminus were essential for radial growth and seemed to support the NF KSP phosphorylation hypothesis of radial growth.

 A direct test of this hypothesis was performed by expressing a KSP phosphoincompetent variant of NF-M in mice. Site-directed mutagenesis was utilized to mutate all KSP serine residues to alanine preventing KSP phosphorylation (NF-M^S→A^ mice) without removing the entire C-terminus of NF-M [9] (Figure 2(c)). Surprisingly, axonal diameter was not altered in NF-M^S→A^ mice suggesting that KSP phosphorylation was not an essential component of radial axonal growth [9] (Figure 2(c)). When taken together with the results obtained from NF-M^TailΔ^ mice, these results suggested that the C-terminus of NF-M mediated radial growth by a mechanism that did not require KSP phosphorylation. Therefore, the precise mechanism by which NF-M C-terminus mediates radial growth has yet to be determined.

## 3. Proximal to Distal Appearance of Phospho-Epitopes of NFs during Axonal Transport

Although NF phosphorylation does not directly regulate radial growth, evidence suggested a progressive appearance of phosphoepitopes [7, 24, 25] as NFs are transported from the neuronal cell bodies to the axons [26]. Radiolabeling of NFs in retinal ganglion cells of mice suggested that NFs were more heavily phosphorylated in distal axonal regions [25]. Moreover, the appearance of phosphoepitopes on NF proteins occurred at distinct developmental stages. NF-H phosphorylation resulted in the appearance of a phosphoepitope that was recognized by the SMI-34 monoclonal antibody [27]. As NFs enter the optic nerve, this epitope was readily visualized suggesting amino acid phosphorylation that contributes to the SMI-34 epitope occurred in neuronal cell bodies [28]. A phosphoepitope recognized by SMI-31 on NF-H was detected only within axons of the optic nerve subsequent to the SMI-34 epitope [28], and the phosphoepitope recognized by RT97 antibodies was detected last and was only evident in axons of optic nerve [28]. Appearance of the RT97 epitope coincided with local accumulation of NFs and initiation of radial growth [28]. Taken together these results suggested that temporally distinct phosphorylation events of NF proteins regulated NF transport in optic nerve axons.

## 4. Multiple Causes of Aberrant NF Accumulations

NF accumulations are hallmarks of neurodegenerative diseases (NDDs). However, it is unclear how NDDs alter NF dynamics resulting in the observed accumulations. Analysis of NFs suggests several potential sites where disease-induced alterations could result in NF accumulations. One such mechanism is disrupted NF transport and altered local accumulation caused by altered phosphorylation dynamics.

Mechanistically, phosphorylation may regulate NF transport by altering NF association with molecular motors. Phosphorylation of NF-H and NF-M altered NF association with kinesin [29, 30] and dynein [26]. Decreased NF-H association with the anterograde motor, kinesin, coincided with the appearance of the C-terminal RT97 phopho-epitope [31] and correlated with increased NF-H association with the retrograde motor, dynein [26]. Taken together, these data suggest that increased C-terminal (KSP) phosphorylation directed the overall flow of NF transport towards neuronal cell bodies providing evidence for a role of altered phosphorylation dynamics in the appearance of phosphorylated NF accumulations within motor neuron cell bodies.

Moreover, deletion of either NF-H [19–21] or NF-M [32] increased the rate of NF transport *in vivo* supporting a role for KSP phosphorylation in regulating NF transport. Therefore, increased rates of NF transport may have resulted from increased association of NFs with kinesin, if loss of a single subunit results in a net loss of phosphorylation. Interestingly, deletion of NF C-termini and all KSP repeats did not alter NF transport rates [22, 33, 34]. Unaltered rates of NF transport in mice expressing truncated NFs appeared to contradict a primary role for NF phosphorylation in regulating NF transport. However, truncation of a single subunit resulted in compensatory phosphorylation of the remaining subunit [22, 23]. Thus, phosphodependent regulation of NF transport cannot be completely ruled out until transport is measured in mice simultaneously expressing C-terminally truncated NF-M and NF-H.

NF-L [35] and NF-M [7] were phosphorylated at serine residues located throughout their amino termini, especially serine^55^. Mimicking constitutive phosphorylation by mutating serine residues to aspartate, such as NF-L^S55D^, altered NF assembly and decreased NF transport [35]. Transgenic mice expressing low levels of NF-L^S55D^ developed NF accumulations within neuronal cell bodies in mice as young as 4 weeks old [8]. Additionally, phosphorylation of NF-M at protein kinase A sites within the N-terminus inhibited C-terminal KSP phosphorylation of NF-M [7]. Thus, phosphorylation of NF-L and NF-M on serine residues located within the N-terminus may be a mechanism to delay NF assembly and phosphorylation thereby preventing ectopic accumulation of NFs within neuronal cell bodies.

Altering NF subunit stoichiometry by overexpression of NF-L [36, 37], NF-M [36], or NF-H [38] altered NF phosphorylation dynamics. NF-L and NF-H overexpression resulted in phosphorylated NF accumulations in motor and sensory neuron cell bodies that were not observed in control littermates [37, 38]. NF-M overexpression resulted in decreased NF-H phosphorylation and increased NF-L expression [36]. Furthermore, NF-L or NF-H overexpression resulted in morphological abnormalities, cell body swelling, and muscle atrophy that were similar to the pathologies observed in amyotrophic lateral sclerosis (ALS) [39, 40]. Neuronal pathology was alleviated in NF-H transgenic mice by simultaneous overexpression of NF-L [41] highlighting the importance of regulating the relative stoichiometry of the individual NF subunit proteins. Altered subunit stoichiometry has been observed in NDDs. NF-L mRNA was selectively decreased in Alzheimer's disease (AD) [42]. NF-L mRNA expression was also reduced in sporadic [43] and superoxide dismutase 1-linked familial ALS [44]. Deletion of NF-L in mice resulted in accumulation of NF-H and NF-M in neuronal cell bodies [45]. If reductions in NF-L mRNA expression lead to reduced NF-L protein, then, as observed in genetically altered mice, altering NF subunit stoichiometry may contribute to NF accumulations observed in both AD and ALS.

A potential mechanism for regulating NF content within the axon is degradation by the ubiquitin proteasome system (UPS) [46–48]. Disruption of two components of the UPS, tripartite motif protein 2 (Trim2^GT^) mice [46] and ubiquitin-specific protease 14 (Usp14) (ax^J^ mice) [49–51], resulted in phenotypic ataxia resulting from motor neuron dysfunction. Upon further examination, Trim2^GT^ mice displayed alterations associated with neurodegeneration [46]. One-month-old Trim2^GT^ mice contained axonal swellings and NF accumulations in regions that overlapped calbindin D-28K immunoreactivity suggesting that the axons originated from Purkinje neurons [46]. By 5 months, Purkinje cell loss was readily apparent. Trim2^GT^ mice showed an 85% reduction in the number of Purkinje cells relative to control mice [46].

 Ax^J^ mice demonstrated a selective loss of large caliber axons within the fourth and fifth lumbar (L4-L5) motor roots [49]. Furthermore, Ax^J^ mice displayed severe neuromuscular junction (NMJ) defects such as swollen and poorly arborized nerve terminals that demonstrated sprouting, denervation, and disorganized postsynaptic AChRs [49]. Within the tibialis anterior (TA) muscle, axonal swellings contained NF accumulations that were observed as early as P7 [49]. Moreover, NMJ defects were accompanied by defects in synaptic transmission. Ax^J^ mice had a 1.5-fold increase in miniature end-plate potential (mEPP) amplitude. Additionally, mEPP frequency and vesicular quantal content were both reduced by ~40% suggesting synaptic vesicle disorganization [49, 50].

Disruptions to both components of the UPS resulted in axonal swellings containing phosphorylated NF accumulations. Trim2 expression was almost exclusively limited to the nervous system in Trim2^GT^ mice [46]. Moreover, restoration of neuronal Usp14 levels (Thy1-Usp transgene) in ax^J^ mice alleviated phenotypic ataxia [52] and NF accumulations [49]. Therefore, altering the neuronal UPS resulted in NF accumulations that were similar to those observed in mice expressing NFs that mimic constitutive phosphorylation within the N-terminal head domain, mice with altered NF stoichiometry, and many human NDDs.

## 5. Is NF Phosphorylation a Mechanism to Enhance Stability by Reducing NF Degradation?

NFs are long-lived axonal proteins whose accumulation within the axon is strictly regulated. Although evidence suggests that NFs associate with molecular motors [26, 29, 30] in a phosphodependent manner to reach their destination, little is known about the mechanisms that prevent excessive NF accumulation and regulate local NF turnover. The appearance of NF accumulations in axons with UPS defects suggests that the UPS has a critical role in the regulation of NF turnover.

Ubiquitin epitopes have been identified on NF-M purified from spinal cord verifying that ubiquitination is indeed NF PTM [48]. Furthermore, NF-L has been identified as a target of the E3 enzyme, Trim2 [46], and NF-M has been identified as a target of the E3 enzyme, carboxyl-terminus of Hsc70 interacting protein (CHIP) [47]. Identification of NF E3 enzymes supports the claim that NFs may undergo ubiquitin-mediated degradation. However, the mechanisms responsible for regulating NF ubiquitination have yet to be determined. Trim2 and CHIP are classified as E3 ligases [46, 47] which are proteins that catalyze the transfer of ubiquitin to substrate proteins [53]. Both Trim2 [54] and CHIP [55] targeted and ubiquitinated protein substrates in a phosphodependent manner. The E3 ligase, c-Cbl, had a similar phosphodependence for its interaction with epidermal growth factor receptor (EGFR) and sprouty-2 (Spry2) [56]. Interestingly, increased phosphorylation of EGFR or Spry2 reduced c-Cbl substrate binding [56]. Although evidence suggested that Trim2 and CHIP require substrate phosphorylation [54, 55], it is unknown if increased phosphorylation would negatively regulate TRIM2 and CHIP affinity for NF subunits similar to c-Cbl. However, evidence suggested that NF stability is increased with increasing phosphorylation. NF phosphorylation was increased in cells [57] and brain slices [58] treated with the phosphatase inhibitor, okadaic acid, resulting in increased NF levels [57, 58]. Furthermore, treating purified NFs with alkaline phosphatase resulted in dephosphorylated NFs that were degraded more rapidly by the calcium dependent protease, calpain, when compared to phosphorylated NFs [59]. Therefore, increased NF phosphorylation may be a mechanism for enhancing NF stability by preventing NF turnover by UPS or protease-dependent mechanisms.

Ubiquitination of the type I/II intermediate filaments, keratins, may offer insight into the interaction between NF phosphorylation and ubiquitination. Like NFs, keratins contain a consensus sequence (^69^QSLLSPL^75^) that is phosphorylated. A proline to leucine mutation at position 74 of keratin 8, K8^P74L^, eliminated the phosphorylation of this consensus site and increased K8 ubiquitination [60]. Similarly, K8^P74A/D^ mutations led to decreased phosphorylation and increased K8 ubiquitination [60]. Conversely, a K8^L71P^ mutation increased K8 phosphorylation and decreased K8 ubiquitination [60]. Taken together these data suggest that phosphorylation and ubiquitination of K8 are reciprocally regulated [61, 62]. The similarity between NFs (type IV intermediate filaments) and keratins (type I/II intermediate filaments) suggests that NF KSP phosphorylation could regulate subsequent PTMs in a manner similar to keratin phosphomotifs.

## 6. NF Phosphorylation Is Increased in Neurodegenerative Diseases

In many NDDs, accumulations of phosphorylated NFs are hallmarks of pathology. Increased phosphorylation resulted in increased NFs [57, 58] and decreased K8 ubiquitination [60], whereas dephosphorylation of the most heavily phosphorylated NF subunit, NF-H, increased its turnover sixfold [59]. Furthermore, NF-L [46] and NF-M [47] have recently been identified as E3 ligase targets. Taken together, these data suggest that ectopic phosphorylation of NFs results in NF accumulations, which may lead to further phosphorylation subsequent to aggregate formation. Delineating the mechanisms that regulate NF phosphorylation may prove to be critical in regulating NF accumulations in NDDs. In this section, we focus on three NDDs as models of NF contribution to disease pathogenesis.

### 6.1. Amyotrophic Lateral Sclerosis (ALS)

ALS is an adult-onset NDD that selectively kills upper and lower motor neurons. Despite common pathogenic features, there is no identifiable genetic linkage in 90–95% of ALS cases (sporadic ALS) while 5–10% of ALS occurrences result from dominantly inherited genetic mutations (familial ALS) [63]. Dominant missense mutations in the gene for the cytoplasmic Cu/Zn superoxide dismutase 1 (SOD1) are responsible for 20% of familial ALS cases [64]. Motor neuron cell bodies contain NF accumulations in cases of sporadic [39] and familial ALS [65].

Mice expressing SOD1-linked mutations resulted in motor neuron pathology that included ectopic accumulations of phosphorylated NFs within lumbar motor neuron cell bodies [63]. Furthermore, SOD1-linked ALS mouse models demonstrated impaired slow axonal transport of NFs as the earliest indication of pathology [66]. In order to identify the role of NFs in SOD1-linked ALS pathogenesis, SOD1^G85R^/NF-L^−/−^  mice were generated [45]. The absence of assembled NFs in axons (NF-L^−/−^) alleviated the selective motor neuron vulnerability and slowed SOD1^G85R^-mediated toxicity. Furthermore, elimination of NF KSP motifs via single or double truncation of NF-M and NF-H C-termini delayed disease onset and extended survival of the SOD1^G37R^ transgenic mouse model [67]. Interestingly, delayed onset and enhanced survival were additive with truncation of both NF-M and NF-H C-termini enhancing survival better than truncation of a single NF C-terminus [67]. Furthermore, preventing NF KSP phosphorylation through C-terminal truncation of NF-M and NF-H enhanced motor neuron survival in SOD1^G37R^ mice [67]. These results suggested that the NF network, particularly NF-M and NF-H C-termini and their phosphorylation, contributes to ALS pathogenesis. However, the mechanism by which removal of axonal NFs or preventing their C-terminal phosphorylation ameliorated ALS pathology remains to be determined.

### 6.2. Charcot-Marie-Tooth (CMT)

**Figure 3:**
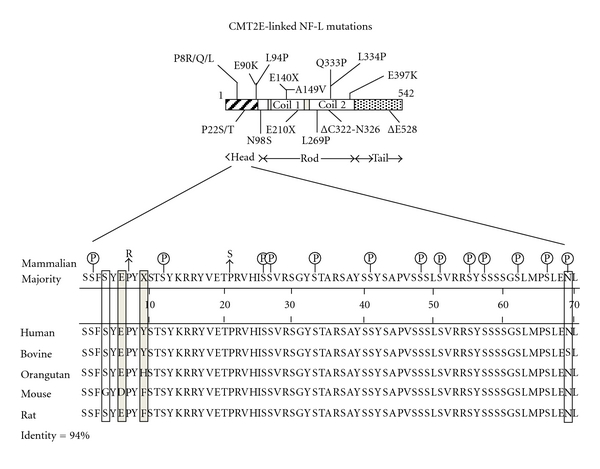
Relative positions of CMT2E-linked NF-L mutations and NF-L head domain phosphorylation sites. (A) Identity and location of all identified CMT2E-linked NF-L mutations. (B) Aligned protein sequences of human, bovine, orangutan, murine, and rat NF-L N-terminal head domains. Identified serine phosphorylation sites within bovine NF-L are indicated along the consensus sequence. Shaded boxes indicate conserved sequence variations, and empty boxes identify nonconserved sequence variations. An X in the consensus sequence identifies amino acid positions that lack an overall consensus between the five species. Sequence homology is indicated by percent identity. CMT2E-linked head domain mutations NF-L^P8R^ and NF-L^P22S^ are identified with an arrow with the corresponding amino acid substitution. These two mutations are located adjacent to phosphorylation sites. Notice that all but one (ser^69^) bovine phosphorylation site is conserved between species. Sequence accession numbers used to generate this figure are as follows: Human NP_006149, Bovine NP_776546, Orangutan NP_001126494, Mouse NP_035040, and Rat NP_113971.

CMT is the most commonly inherited peripheral neuropathy. Based upon the specific genetic defect CMTs are grouped into four main types, CMT1-4 with each type having several subtypes [68, 69]. CMT2E has been linked to mutations throughout the functional domains of NF-L [70] (Figure 3(A)). NF-L mutations are typically autosomal dominant though two recessive forms have been identified [71, 72] (Figure 3(A)). CMT2E recessive mutations introduce a nonsense mutation resulting in loss of NF-L protein.

Two CMT2E-linked NF-L mutations are located at positions, Pro^8^ and Pro^22^. These mutations are located adjacent to known bovine head domain phosphorylation sites, Ser^2^, Ser^12^, Ser^26^, and Ser^27^(Figure 3(B)). Interestingly, Pro^8^ and Pro^22^ along with all N-terminal serine residues are highly conserved across mammals [73] (Figure 3(B)). The bovine NF-L head domain contains thirteen phosphorylation sites [73]. All but one of these sites (Ser^69^) have complete sequence homology in NF-L from a variety of mammals [73] (Figure 3(B)).

When expressed in cultured cells, the hNF-L^P22S/T^ mutation abolished NF-L head domain phosphorylation [74]. Additionally, mutation of a proline residue in keratin 8 prevented phosphorylation of a nearby serine residue [60]. Taken together, these data suggest that NF-L^P8R^ and NF-L^P22S^ mutations may prevent N-terminal phosphorylation. As previously discussed, expressing NF-L that mimics constitutive phosphorylation of an N-terminal serine, NF-L^S55D^, prevented NF assembly [75], and N-terminal phosphorylation of NF-M prevented C-terminal phosphorylation [7]. Based on the previously described role of NF phosphorylation, abolished N-terminal phosphorylation would result in ectopic NF assembly and phosphorylation [7, 8, 75], altered NF transport [21, 26, 30, 33, 35], and ectopic NF accumulations [7, 8, 75]. Consistent with this prediction, transgenic mice expressing the CMT-linked head domain mutation, hNF-L^P22S^, displayed disrupted axonal transport [76]. Furthermore, mutations within a conserved sequence at the end of the rod domain, hNF-L^E397K^, also resulted in ectopic NF phosphorylation as early as 1 month [77].

### 6.3. Spinal Muscular Atrophy (SMA)

SMA is an autosomal recessive disorder that is the leading genetic cause of infantile death [78]. SMA is caused by a deficiency in survival motor neuron (SMN) protein levels produced by the *SMN1 *gene [79, 80], which is a ubiquitously expressed protein that has a well-described role in RNA metabolism [81–85]. Deficiencies in SMN protein levels lead to skeletal muscle paralysis [86]. The severity of SMA is dependent on the relative copy number of the *SMN2* gene which produces ~15% functional SMN that compensates for the loss of *SMN1 *[87, 88]. As a result, SMA has a broad disease spectrum made up of SMA type 0, I, II, III, and IV [89].

Within motor neurons, SMA patients [90] developed phosphorylated NF accumulations within NMJs. Similar NF accumulations were one of the earliest pathological alterations observed in SMA mouse models [91–94]. The cause and pathogenic properties of NF accumulations are poorly understood in SMA. However, recent work demonstrated that NF accumulations were likely a result of local NF alterations [95]. Ectopic NF accumulations within motor neuron cell bodies were not apparent early or late in disease [91]. Moreover, the NF network was not altered in proximal segments of motor axons of the fifth lumbar ventral root [95], which is one of three lumbar segments that together form the sciatic nerve. Interestingly, mice with alterations to the UPS displayed phosphorylated NF accumulations [49] strikingly similar to those observed in SMA models [90–93]. Taken together, these results suggest that mechanisms disrupting NF turnover within motor neurons may be responsible for NF accumulations within NMJ of SMA patients. One potential NF alteration that could lead to reduced NF turnover and subsequent accumulation is altered NF phosphorylation.

## 7. Conclusions

NFs are abundant cytoskeletal proteins that undergo various PTMs. The most abundant PTM is phosphorylation of NF subunit proteins within myelinated axons. Phosphorylation of NFs was initially documented nearly 30 years ago and thought to regulate radial growth within myelinated axons. However, a series of recent analyses conducted on genetically modified mice has provided evidence against the role of NF phosphorylation in radial growth. Current evidence suggests that NF phosphorylation is both spatially and temporally regulated, which may be a mechanism to regulate NF assembly and accumulation. The presence of phosphorylated NFs in human NDDs suggests that altered NF phosphorylation dynamics may contribute to aberrant NF accumulation. Therefore, understanding the role of and mechanisms regulating NF PTM may prove critical to our understanding of the development and functioning of the nervous system in both healthy and diseased neurons.
